# Images in haematology: Primary cutaneous diffuse large B cell lymphoma, leg type with an unusual presentation: A diagnostic challenge

**DOI:** 10.1002/jha2.647

**Published:** 2023-01-31

**Authors:** Pablo Villagrasa‐Boli, Mar Garcia‐Garcia, Luis Ignacio Sancho‐Val, Pedro Paul, Ana Leticia Tardin‐Cardoso, Mariano Ara‐Martín, Lucía Prieto‐Torres

**Affiliations:** ^1^ Dermatology Department Lozano Blesa University Hospital Zaragoza Spain; ^2^ Health Research Group GIIS100 of Health Research Institute IIS Zaragoza Spain; ^3^ Pathology Service, Lozano Blesa University Hospital Zaragoza Spain; ^4^ Department of Pathology and Surgery. Faculty of Medicine University of Zaragoza Zaragoza Spain; ^5^ Haematology Service Lozano Blesa University Hospital Zaragoza Spain; ^6^ Nuclear Medicine Department Lozano Blesa University Hospital Zaragoza Spain

**Keywords:** cutaneous lymphoid, dermatology, lymphoid malignancies, pathology

1

An 83‐year‐old woman with a history of arterial hypertension treated with amlodipine, and a well‐differentiated cutaneous squamous cell carcinoma completely excised during the past year, was referred to dermatology consultations for the progressive appearance of indurated nodular skin lesions over the last two months, which began on the right lower extremity and progressed to the upper extremities, trunk and buttocks, with no other symptoms associated. With the differential between cutaneous lymphoma versus subcutaneous metastases, a punch biopsy of one of the lesions was performed. Histopathological examination demonstrated a dense hypodermal infiltrate composed of atypical large lymphocytes showing a round‐shaped nucleus with vesicular chromatin and prominent single or multiple nucleoli. The immunohistochemical study showed positivity in neoplastic cells for CD79a, CD20, Bcl‐2, MUM‐1, c‐myc and IgM, along with a high expression of Ki‐67; and negativity for CD3, CD10, Bcl‐6, CD30, p53, PD‐L1 and Epstein‐Barr Virus Encoded RNA. Molecular analyses were then performed without detecting BCL‐2, BCL‐6 or c‐MYC rearrangements by fluorescence in situ hybridization, as well as the absence of p.L265P mutation in MYD88 through a quantitative polymerase chain reaction.

Taking into account both histopathological and clinical findings, a primary cutaneous diffuse large B cell lymphoma, leg type (PCDLBCL‐LT) and skin involvement in the course of a systemic DLBCL were considered, so blood tests and a positron emission tomography (18‐FDG PET/CT) were then solicited.

Blood tests showed a haemoglobin value of 12.5 g/dl with a normal white blood cell count and a slight β‐2‐microglobulin elevation (3.33 mg/L) in the context of a decreased renal function (54.38 ml/min/1.73m^2^).

18‐FDG PET/CT revealed 23 hypermetabolic subcutaneous nodules distributed predominantly in the right arm and lower extremities, with no sign of disease in other locations (Figure 1), confirming the primary cutaneous origin of the disease and the final diagnosis of a PCDLBCL‐LT.

**FIGURE 1:**
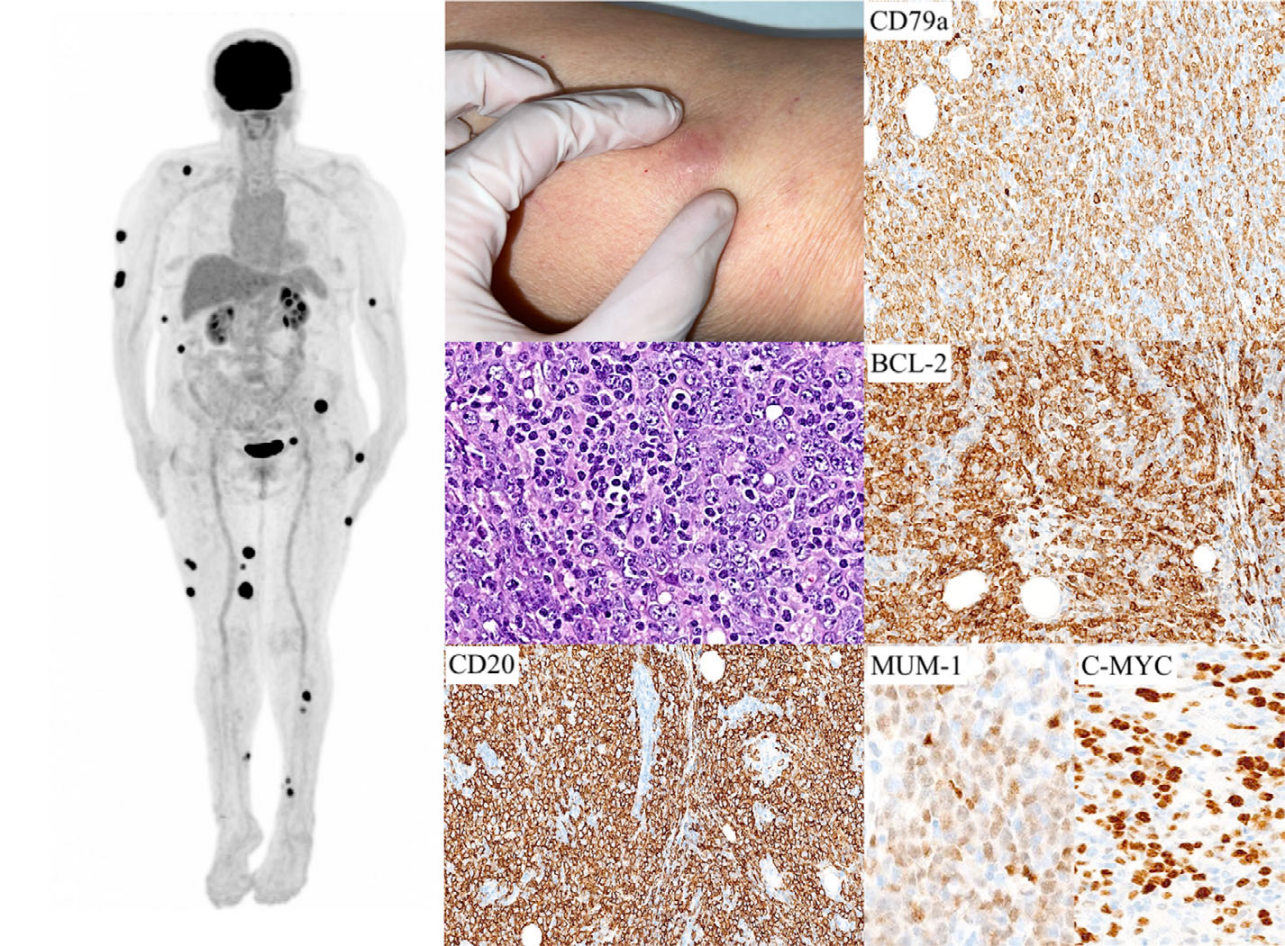
Several images showing positron emission tomography (18‐FDG PET/CT) results, the clinical appearance of one of the lesions located on the leg, and histopathological results of the skin biopsy with immunochemistry markers indicated for each picture

The patient underwent treatment with 6 cycles of rituximab, cyclophosphamide, doxorubicin, vincristine and prednisone, with progressive improvement of the skin lesions and complete resolution of them, and is currently being followed up by haematology and dermatology departments.

Although the majority of PCDLBCL‐LT, arise in the lower limbs (80%–85%), some patients can develop the disease in other areas, but in those cases, the lesions are usually individual or few in number, contrary to our case. PET imaging tests have a major role in the initial staging of the disease and treatment response evaluation, and they could even avoid bone marrow biopsy in selected patients. The double expression of bcl‐2 and c‐myc is frequent in PCDLBC‐LT, although the prognostic value of this dual expression is yet unclear, whereas the absence of the MYD88 mutation could be associated with both better response to lenalidomide as well as better prognosis. For these reasons, immunohistochemical and molecular techniques can be helpful in defining prognosis and deciding therapeutic options in patients with PCDLBCL‐LT and should be performed in routine practice.

In conclusion, we present an extremely rare 18‐FDG PET/CT image of a PCDLBCL‐LT with 23 subcutaneous nodules, underlying the importance of this test for the study of these patients.

## AUTHOR CONTRIBUTIONS

Lucía Prieto‐Torres – Attending the case and performing clinical research and manuscript revision.

2

Pablo Villagrasa‐Boli – Attending the case, revising existing literature and manuscript writing.

3

Luis Ignacio Sancho‐Val – Attending the case and manuscript revision.

4

Pedro Paul – Attending the case and manuscript revision.

5

Ana Leticia Tardin‐Cardoso – Performing the imaging tests and their clinical interpretation.

6

Mar García‐García – Pathology diagnosis, taking microscopic images and manuscript revision.

7

Mariano Ara‐Martín – Proof checking.

## CONFLICT OF INTEREST

The authors declare that they have no conflict of interest.

## FUNDING INFORMATION

No funding has been received for this article.

